# Review: Digital experiences and their impact on the lives of adolescents with pre‐existing anxiety, depression, eating and nonsuicidal self‐injury conditions – a systematic review

**DOI:** 10.1111/camh.12619

**Published:** 2022-12-07

**Authors:** Katarzyna Kostyrka‐Allchorne, Mariya Stoilova, Jake Bourgaize, Miriam Rahali, Sonia Livingstone, Edmund Sonuga‐Barke

**Affiliations:** ^1^ School of Academic Psychiatry, Institute of Psychiatry, Psychology and Neuroscience King's College London London UK; ^2^ Department of Media and Communications London School of Economics and Political Science London UK; ^3^ Department of Child & Adolescent Psychiatry Aarhus University Aarhus Denmark

**Keywords:** Adolescent mental health, emotional disorders, digital experiences, social media

## Abstract

**Background:**

Published systematic reviews provide evidence linking positive and negative digital experiences to adolescent mental health. However, these reviews focus on the general public rather than the digital experiences of adolescents with different pre‐existing mental health conditions and so may be limited in their clinical relevance. We review publications relating to anxiety, depression, eating disorders and nonsuicidal self‐injury to identify common and condition‐specific digital experiences and how these may be implicated in the origins and maintenance of these mental health conditions.

**Methods:**

A systematic literature search using a combination of mental health, digital experience (including social media use), and age of the target population terms was conducted on four databases. Detailed findings from the included studies were summarised using a combination of thematic and narrative methods.

**Results:**

Five qualitative and 21 quantitative studies met the eligibility criteria for inclusion (*n* = 5021). Nine studies included adolescents with depression, one with eating problems, two with nonsuicidal self‐injury and 14 with multiple emotional health conditions. The review identified six themes related to the target populations' digital experiences: (a) social connectivity and peer support; (b) escape and/or distraction; (c) social validation and social comparison; (d) accessing/creation of potentially harmful content; (e) cyberbullying; and (f) difficulties with self‐regulation during engagement with digital media.

**Conclusions:**

Digital practices of adolescents with pre‐existing clinical vulnerabilities are complex and encompass a range of positive and negative experiences, which appear to have common elements across different clinical populations. The literature is currently too limited to identify disorder‐specific practices, with too few direct or indirect comparisons between conditions.

**Key practitioner message**

- Digital technologies have redefined how adolescents learn, work, play and socialise. These digitally related changes in adolescent lifestyles coincide with a consistent trend of an increase in adolescent mental health problems.
- The digital world provides psychologically vulnerable adolescents with opportunities for social connection and support and allows distraction from their mental health problems.
- However, some young people find it difficult to self‐regulate their digital engagement and their digital experiences can exacerbate psychological difficulties or lead to the normalisation of pathological behaviour.
- Future research should be designed in a way to better understand the more nuanced impact of a multiplicity of digital experiences and how these might differ for specific clinical groups.

## Background

Studies comparing data from population cohorts collected at different points across the last two decades report increases in the prevalence of adolescent mental health problems such as anxiety and depression (Collishaw, 2015; Keyes, Gary, O'Malley, Hamilton, & Schulenberg, 2019; Patalay & Gage, 2018; Thorisdottir, Asgeirsdottir, Sigurvinsdottir, Allegrante, & Sigfusdottir, 2017), eating problems (Neumark‐Sztainer, Wall, Eisenberg, Story, & Hannan, 2006; von Soest & Wichstrøm, 2014; Wu et al., 2022) and self‐harm (Griffin et al., 2018; Patalay & Gage, 2018; Tørmoen, Myhre, Walby, Grøholt, & Rossow, 2020). Researchers trying to explain these increases have noted that they coincide with other socio‐cultural and demographic changes with putative significance as risk factors for poor mental health such as academic and social pressures, family difficulties, a precarious job market and an uncertain future (Cosma et al., 2020; Högberg, Strandh, & Hagquist, 2020; Reiss et al., 2019; Voßemer & Eunicke, 2015).

One major change over this period relates to how digital technologies have revolutionised the ways that adolescents learn, work, play and interact with each other. For instance, nine in 10 children in the UK own a smartphone by the time they reach the age of 11 years (Ofcom, 2022). The large majority of children aged 11 use social media (78%) and have a social media profile (72%) despite being younger than the minimum age requirement (note that it is not always necessary to have a social media profile to view content posted as ‘public’). This rises to nearly all children aged 17, 97% of whom use social media and 94% have a profile (Ofcom, 2022). The picture is similar in other countries (Smahel et al., 2020). Given the coincidence between the increase in adolescent mental health problems and these dramatic, digitally related changes in lifestyle, it is no surprise that much research has focused on the relationship between digital experiences and adolescent mental health.

The digital world has been described in terms of its relationship to adolescents' mental health as a triple‐edged sword (Hollis, Livingstone, & Sonuga‐Barke, 2020). First, it provides great opportunities for personal growth and development. Second, it exposes adolescents to inappropriate content and adverse experiences that place them at risk. Third, it could be harnessed for the identification and treatment of mental health problems. These benefits, risks and opportunities of the digital world for mental health are generally supported by recent reviews (Hollis et al., 2017; Odgers & Jensen, 2020; Orben, 2020). However, the conclusions that could be drawn from such reviews have been constrained by methodological and measurement limitations of many published studies – in particular, the dominance of cross‐sectional designs and the lack of studies of risks and benefits that distinguish between different types of digital engagement. Thus, while many studies report a positive, if weak, correlation between screen time and mental ill‐health, the direction of causation, the validity of the measures and the importance of confounding factors remain contested (Orben, 2020).

The relevance of these reviews, and the studies on which they are based, to clinical practice is limited by a lack of focus on the nature of the digital experiences of, and impacts on, young people with pre‐existing diagnosed mental health conditions. Additionally, few if any studies have directly compared the experiences of adolescents with different conditions. Hence key questions remain unanswered. Do adolescents with pre‐existing mental health conditions differ in terms of why and how they engage with the digital world compared to peers without such difficulties? Are specific mental health conditions linked to different patterns of digital usage? What role do such differences play in the development and escalation/maintenance of these conditions?

Here we report the results of a comparative systematic review of the published literature on digital engagement and its impact (positive and negative) on adolescents with four different types of pre‐existing clinically significant mental health difficulties: anxiety, depression, eating problems and nonsuicidal self‐harm. These were selected because of prior research suggesting a role for digital experiences both in the development and maintenance of these difficulties (Stoilova, Edwards, Kostyrka‐Allchorne, Livingstone, & Sonuga‐Barke, 2021). The nature of those digital experiences and exposure is often obscured in systematic reviews focused on screen time (e.g. length of exposure to any kind of screen content or interaction). However, the growing research consensus is that what matters more than time is the nature, quality and perceived meaning of digital engagement, given that screen time may involve, for example, viewing self‐harm content or seeking help for one's mental health. More broadly, digital engagement may enable opportunities (e.g. gaining health information, professional help or social support) or risks (e.g. encountering cyberbullying or images of self‐harm or proanorexia communities), although there can be overlap and ambiguities in making this distinction. Since little progress has been made in identifying just which qualities of digital engagement matter, particular attention is paid in the review to measures of digital engagement and the effects they reveal.

## Methods

We conducted a systematic evidence review (Gough, Thomas, & Oliver, 2012; Sutherland, 2004) using the Preferred Reporting Items for Systematic Review and Meta‐Analysis (PRISMA) protocol (Moher et al., 2015). The review aimed to answer the following research questions:

1. Are digital experiences different for adolescents with pre‐existing clinically significant anxiety, depression, eating problems and nonsuicidal self‐injury?
2. Are adolescents with these conditions at greater risk of harm from digital engagement compared to those in nonclinical groups? Are there differences between different conditions?
3. What are the potential benefits of digital engagement for adolescents with these conditions?
4. How can studies of digital engagement in adolescents with emotional conditions inform clinical practice?

Four databases, Embase, Medline, PsycInfo and Web of Science, selected for their relevance to the topic and aims, were searched in February 2022 for articles in the English language published during the past 10 years (2012 to current). The searches were limited to the past 10 years to reflect the substantial changes in digital technologies that occurred during this time (e.g. the rapid rise of platforms such as Instagram and TikTok). The year 2012 also marked an important shift in the way young people used digital media, with the switch to personal devices, and 12–15‐year‐olds started using the internet as much as watching television (Ofcom, 2012).

The search protocol involved a combination of terms on: (a) mental health conditions (specifically, eating disorders, self‐harm, depression, anxiety), (b) digital experience, and (c) the age group of the target population. A detailed list of the search terms is provided in the Appendix S1. The research protocol was registered on PROSPERO (CRD42022318672).

For inclusion in the review studies had to:

- Report primary data analysis of quantitative or qualitative empirical research.
- Include only participants who were adolescents with a mean age of 12–19 years or, for longitudinal research, were younger than 20 during the final wave of data collection.
- Report findings separately for groups with clinically significant depression and/or anxiety and/or nonsuicidal self‐injury and/or eating disorders. That is, they had a diagnosis, were seeking/receiving help or were in contact with clinical services regarding any of these mental health conditions.
- Report data on digital experiences with social media and/or gaming and/or internet and/or web chat and/or web forum use and/or mobile phone/smartphone use.
- Be from a peer‐reviewed article, a book or peer‐reviewed chapter, conference proceedings, a report, a case study, a clinical commentary or a clinical position paper.

Studies were excluded if:

- They were reported in a PhD thesis, book review, poster or conference abstract.
- They were a meta‐analysis or a review (systematic, scoping, narrative).
- Digital engagement was measured only with ‘screen time’, that is the duration of use.
- Clinical status was based only on symptom counts (i.e. there was no indication of any clinical contact or diagnosis).

Given the breadth of our remit (including concerning implications for clinical practice) and the anticipated shortage of relevant published papers, we included a wide range of studies reporting different types of evidence in the review. These encompassed cross‐correlation and longitudinal correlation studies, case‐control studies, qualitative analyses of interviews and focus groups and clinical guidelines and recommendations. All titles and abstracts were reviewed independently by one of three researchers using a traffic‐light system to mark the studies as ‘include’, ‘unsure’ or ‘exclude’. All studies coded as ‘unsure’ were screened by a second reviewer. The full texts were screened using the same process. Additionally, a fourth reviewer independently assessed 25% of the potentially eligible full texts. Any disagreements during the reliability check or regarding studies coded as ‘unsure’ were resolved during a group discussion.

The final sample of the studies that met the eligibility criteria was subjected to a review‐specific quality appraisal and relevance assessment using a Weight of Evidence (WoE) framework (Gough, 2007). Each study was given a score of 1 = poor, 2 = fair and 3 = good for three sets of criteria: (a) WoE A: Score 1–3 for general quality and execution of the study; (b) WoE B: Score 1–3 for mental health measures and analysis; (c) WoE C: Score 1–3 for digital engagement measures and analysis (see Table 1 for details).

**Table 1:** Weight of evidence assessment criteria used during the review process

Area of appraisal	Poor = 1	Fair = 2	Good = 3
General quality and execution of the study	Mixes adolescents and adults in reporting results. Design and methods are not appropriate to answer the research question. No clear hypotheses or research questions. No clear links between methods and findings. No discussion of the relationship between mental health and digital engagement (pathways). No report of descriptive statistics and correlations or (for qualitative studies) insufficient discussion.	Reports data on adolescents separately. Design and methods appropriate to answer the research question. Clear hypotheses and/or research questions. Clear links between methods and findings. Reports descriptive statistics and correlations coefficients or, for qualitative studies, offers a sufficient discussion.	Reports data on adolescents separately. Design and methods appropriate to answer the research question. Clear hypotheses and/or research questions. Clear links between methods and findings. Comprehensive reporting of the analysis. Statistical methods are appropriate to test the hypotheses. Has a theoretical or statistical model to explain the relationship between mental health and digital engagement or, for qualitative studies, offers a comprehensive discussion.
Mental health measures and analysis	No clear definition of clinical population or a mix of clinical or nonclinical samples. No clear definition measurement of mental health conditions or do not use validated measures. Analysis combines target disorders^a^ with other, nontarget, disorders.	Clearly defined clinical population or only clinical sample. Clear definition and measurement (with a validated instrument) of mental health conditions. Analysis combines multiple target disorders^a^.	Clearly defined clinical population or only clinical sample, or clear comparison between clinical and nonclinical samples. Clear definition and measurement (with a validated instrument) of mental health conditions. Separate analyses on different target disorders^a^.
Digital engagement measures and analysis	Poor measure of digital engagement (e.g. screen time) or using other measures as a proxy (e.g. device ownership).	Clear and established measure of digital engagement. Includes unidimensional^b^ measure of digital engagement. Measures only potentially negative engagement (e.g. exposure to online risks). Does not separate risk from harm.	Includes multidimensional^c^ measures of digital engagement. Measures both potentially positive and negative engagement (e.g. exposure to online risks and opportunities). Measures harm separately from experiences of online risks.

^a^ Target disorders = depression, anxiety, nonsuicidal self‐injury, eating disorders and internalising problems.

^b^ Unidimensional = focused on one dimension of digital engagement only (e.g. social media, gaming, internet, web chat or web forum, mobile phone/smartphone use).

^c^ Multidimensional = measured several different areas of digital engagement.

An overall average score (WoE D) between 1 and 3 was then assigned to each study. Twenty‐five of 26 studies received WoE D scores of 2.0 or above. One article (Hadwiger, Middleman, & Pitt, 2019) received a score of 1.7. However, as it was a clinical case report and the only study that focused on a clinically diagnosed eating disorder, it was retained for data extraction and analysis along with the other 25 articles. The details of each study were extracted using a prespecified data extraction form retaining key information about the study design, measures and findings.

## Results

**Figure 1:**
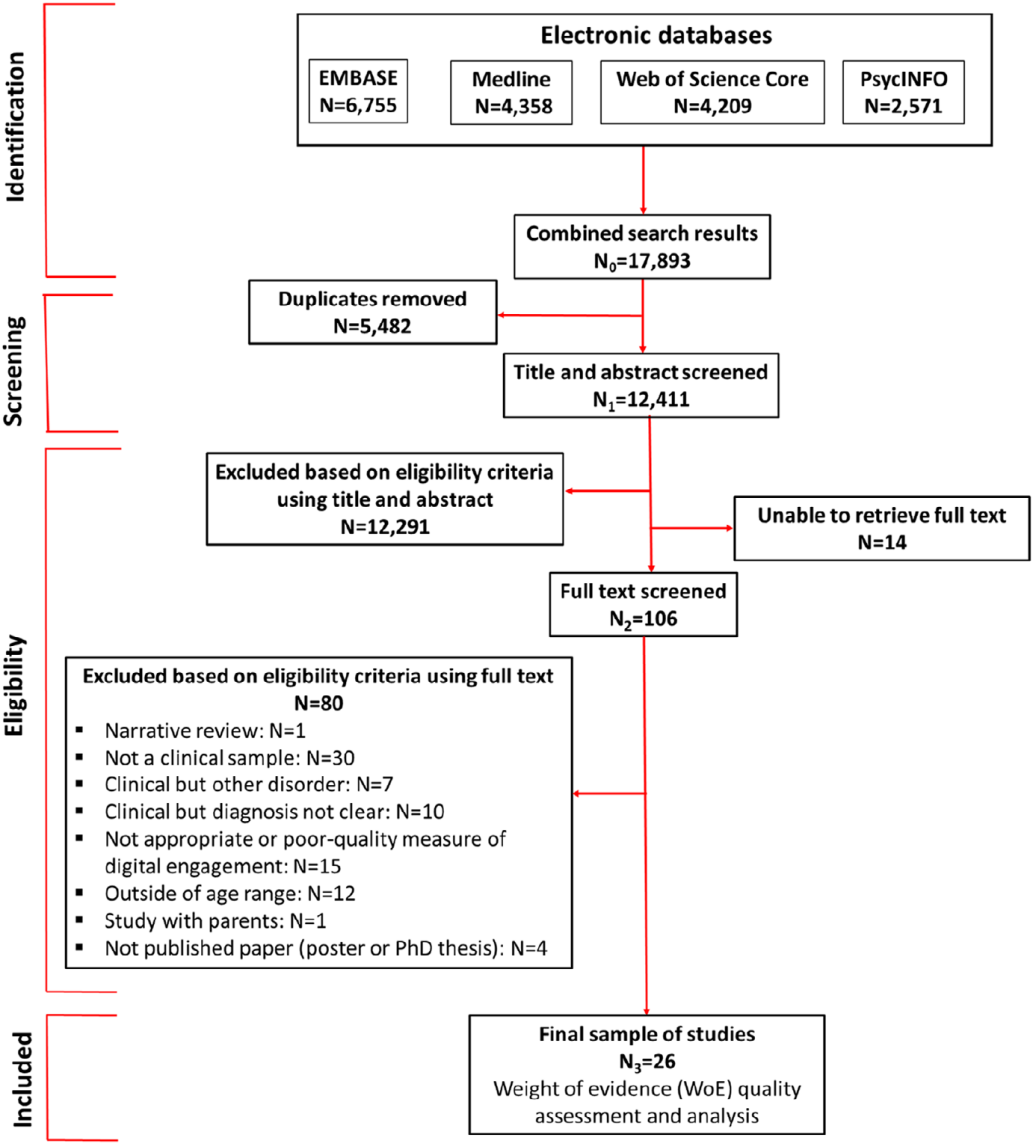
PRISMA diagram

The search yielded 17,893 results and 12,411 remained after the deduplication of records (see Figure 1). A total of 12,291 publications were excluded by applying the eligibility criteria at the stage of title and abstract and 120 studies remained for full‐text screening. Of these, 14 publications could not be accessed and 106 were screened. The most common reasons for exclusion related to the clinical characteristic of the sample: 30 articles did not include a sample with clinical‐level needs, and a further 27 did include a clinical sample but either the information about the type of disorder was not clear (*n* = 10) or adolescents in the sample had a disorder that did not fall within the inclusion criteria (e.g. behavioural or externalising; *n* = 7). Fifteen of the excluded studies did not include a good quality or appropriate measure of digital experience and 12 included participants outside the selected age range. The complete list of reasons for exclusion is provided in Figure 1.

The full‐text review procedure and quality appraisal resulted in 26 articles being retained for analysis. Of these, 13 studies were conducted in the United States, seven in Turkey, and the remaining six took place in China, Hungary, Ireland, Spain, Switzerland and the United Kingdom. The selected articles included five qualitative studies involving 96 participants and 21 quantitative studies, which included 4925 participants. The majority of participants in the included studies were female (62% and 60%, respectively) and were recruited from various settings including inpatient, outpatient and community services. For a breakdown of qualitative and quantitative studies included in the review, see Table 2. None of the included studies adopted a mixed‐method approach. Detailed information extracted from the included studies is provided in Table S1.

**Table 2:** Qualitative and quantitative studies included in the review broken down by mental health condition

Mental health condition	Qualitative studies	Quantitative studies
Depression	Radovic, Gmelin, Stein, and Miller (2017)	Akkin Gurbuz, Demir, Gokalp Ozcan, Kadak, and Poyraz (2017), Alpaslan, Soylu, Kocak, and Guzel (2016)^a^, Cao et al. (2020), Li et al. (2021), Onat, Ozyurt, Ozturk, and Akay (2019)^a^, Sahin and Usta (2020), Ucar et al. (2020)^a^
Nonsuicidal self‐injury	Jacob, Evans, and Scourfield (2017)	Lanzillo, Zhang, Jobes, and Brausch (2021)
Eating problems	Hadwiger et al. (2019)	N/A
Multiple target mental health conditions (e.g. depression, anxiety, nonsuicidal self‐injury, eating disorders, internalising problems).	Weinstein et al. (2021); van Rensburg, Klingensmith, McLaughlin, Qayyum, and van Schalkwyk (2016)	Firat et al. (2018), Gansner et al. (2019), Gansner, Nisenson, Carson, and Torous (2020), Gansner, Nisenson, Lin, Carson, and Torous (2022), Gansner et al. (2022), Martin‐Fernandez et al. (2016); Meszaros, Gyori, Horvath, Szentivanyi, and Balazs (2020)^a^, Mullen, Dowling, and O'Reilly (2018)^a^, Nesi et al. (2021), Nesi et al. (2021), Nesi, Wolff, and Hunt (2019), Ucar et al. (2018), Werling, Walitza, Gerstenberg, Grunblatt, and Drechsler (2022)^a^

^a^ Denotes case‐control studies that compare clinically diagnosed adolescents with adolescents without mental health difficulties. No study focused only on anxiety though anxiety was a focus in studies of multiple conditions.

The review of included studies identified six main themes related to young people's digital experiences. These were: (a) using digital media for social connection, to seek peer support and acceptance (5 studies); (b) using digital media for escape or distraction (4 studies); (c) using digital media for social validation or social comparisons (7 studies); (d) accessing or creating harmful content (7 studies); (e) cyberbullying (5 studies); and (f) self‐regulation of digital engagement, including perceived ‘addiction’ to the internet/digital media (16 studies).

### Using digital media for social connection, to seek peer support and acceptance

Three qualitative studies explored how adolescents use digital media to connect socially and to seek support and acceptance from peers (Radovic et al., 2017; Weinstein et al., 2021). Adolescents described using social media to stay in touch with family and friends, especially, those who were physically distant. They spoke of the relief that comes after sharing difficult emotions online and valued the social support received in response to their help‐seeking posts online. Adolescents emphasised the near‐instant nature of such support and the ability to reach a large group of peers, who provided acceptance and understanding. However, for some adolescents, the issue relating to support was more complex. On the one hand, adolescents found it helpful to know that they were not alone, being one of many struggling with similar psychological difficulties (Radovic et al., 2017). On the other hand, especially in the case of adolescents who self‐harmed, online contact allowed the discovery of new methods, exposed them to ‘triggering’ content (Radovic et al., 2017) or, through receiving acceptance from online peers, led to the normalisation of pathological behaviour (Jacob et al., 2017).

The results of quantitative research provide further information about the extent of social support received online and social connections among adolescents with clinical‐level difficulties. Nesi et al. (2019) reported that 57% of adolescents who were admitted to an inpatient unit reported positive online experiences, such as receiving social support or encouragement, 2 weeks before hospitalisation. The proportion of girls receiving support this way was significantly higher than boys (64% vs. 46%, respectively). Although the results of the study by Akkin Gurbuz et al. (2017) did not show a significant difference between the proportion of depressed and nondepressed adolescents who engaged in social interactions online (e.g. chat online with friends, read their status updates or make friends online), the relationship between the severity of depression symptoms and online engagement may be more nuanced. Using an ecological momentary assessment approach, Gansner, Nisenson, Lin, Carson, and Torous (2022) showed that on days when young people used smartphones less, their depression symptoms were worse. This is consistent with the findings of Cao et al. (2020), which showed that higher depression symptoms in adolescents with a diagnosis of major depressive disorder were negatively correlated with phone call duration and the number of text messages (−.60 < *r* < −.40; *p* < .05, for both smartphone use measures). On the one hand, lower smartphone use may be a sign of social withdrawal and anhedonia that accompany an increase in depressive symptoms. On the other hand, using smartphones and online engagement, more generally, may provide respite from worsening depression. At times, when access to this source of relief is less available, symptoms of depression may feel more severe (Gansner, Nisenson, Lin, Carson, & Torous, 2022).

Overall, the literature suggests that digital media may provide adolescents, who have mental health difficulties, with positive social experiences, instant peer support and acceptance. However, male sex and greater severity of depression symptoms may reduce opportunities for benefiting from these positive digital practices. Some social connections could also exacerbate psychological difficulties and lead to the normalisation of pathological behaviour.

### Coping through escape or distraction

In addition to reporting positive social digital *interactions*, adolescents who took part in the qualitative studies described *solitary* digital practices that helped them to cope with low mood through distraction from their difficulties. These included, for example, listening to music, reading happy or funny quotes, or watching cute animal videos (Radovic et al., 2017; Weinstein et al., 2021). The notion that adolescents with emotional mental health problems use social media to take their minds off things was supported by one quantitative study. Sahin and Usta (2020) reported that the severity of depression symptoms was moderately positively correlated with using social media to escape (*r* = .425, *p* < .05). However, sometimes such a way of coping could be problematic and perpetuate the existing emotional problems. A group of young people with internalising disorders, who mainly used video games to ‘hide themselves away or to avoid discomfort’ also met DSM‐5 criteria for Internet Gaming Disorder (Martin‐Fernandez et al., 2016, p. 129). In contrast, adolescents with coexisting externalising disorders and Internet Gaming Disorder played mainly for entertainment. The authors suggested that using video games as means to escape own psychological difficulties was often linked to more loneliness and physical confinement at home, which, in turn, could worsen family and social functioning.

Overall, findings relevant to this theme are limited. Although solitary digital practices help some adolescents to find distraction and temporary relief from their psychological difficulties, such a way of coping may be maladaptive and could be linked to difficulties in regulating use. Previous research suggests that adolescents' digital literacy is linked to their coping skills in online contexts; improving both of these through targeted interventions could help adolescents to engage in digital practices that improve their mental health and minimise digitally related harm (Vissenberg, d'Haenens, & Livingstone, 2022).

### Social validation and social comparisons

In parallel with describing their positive social interactions and solitary online activities, adolescents acknowledged that some of their digital practices were motivated by seeking approval or acceptance in the form of digital social feedback, such as ‘likes’, ‘hearts’, views, shares or follows (Jacob et al., 2017; Weinstein et al., 2021). This may lead some adolescents to post ‘attention‐seeking’ content, such as images of self‐harm (Jacob et al., 2017) or sexually suggestive photos, or to exaggerate the positive aspects of otherwise mundane events or activities (Radovic et al., 2017). The desire for social validation may also lead some adolescents to carefully curate how their life is represented on social media and, for example, delete posts with too few ‘likes’ to avoid appearing unpopular (Weinstein et al., 2021).

Although participants found it easier to hide their true feelings online compared to face‐to‐face interactions (van Rensburg et al., 2016), the pressure to constantly ‘put up a good front’ on social media was described as ‘exhausting’ and the lack of sufficient or immediate social feedback could result in feeling disappointed or ignored (Weinstein et al., 2021). Moreover, for adolescents, who are feeling sad or down, exposure to overly positive and filtered content could create a false sense that everyone around them is having a better time, is happier or more popular. This, in turn, could lead to feeling inferior, sad or jealous (Radovic et al., 2017; Weinstein et al., 2021) and, ultimately, could exacerbate psychological difficulties.

These findings are corroborated by quantitative research. Negative emotional responses to social media (e.g. including feeling left out and feelings of missing out) were positively correlated with the severity of internalising symptoms (*r* = .43, *p* < .01) in a group of adolescents hospitalised for mental health difficulties (Nesi, Burke, Extein, et al., 2021). Moreover, two weeks before hospitalisation for psychological difficulties, 37% of adolescents compared themselves negatively to others and 31% felt left out or excluded (Nesi et al., 2019). These negative experiences were reported by more females than males (50% vs 17% and 36% vs 22%, respectively). Finally, compared with adolescents with other conditions, adolescents with internalising difficulties reported a more negative assessment of the influence of digital media on their mood and mental wellbeing during lockdown (Werling et al., 2022).

In sum, adolescence is a period of increased need for peer acceptance and social belonging. It is, therefore, not surprising that young people embrace the new opportunities for boosting social popularity afforded by the digital world. However, this may lead to two kinds of negative outcomes for adolescents who have emotional difficulties. First, it may motivate them to engage in risky or problematic online practices to gain positive social feedback or to appear popular. Second, viewing heavily filtered posts that depict only happy and successful events and that are a poor reflection of the more mundane reality could evoke feelings of missing out and lead to negative social comparisons that affect the mood of clinically vulnerable adolescents.

### Accessing and creating harmful content

Three qualitative studies described how young people's digital practices afford opportunities to access what could be broadly termed ‘harmful content’ (Jacob et al., 2017; Radovic et al., 2017; Weinstein et al., 2021). Such content included videos, images or text (or a combination of these) related to self‐harm, disordered eating or depression that could be either perceived as distressing or promoting negative coping. For some adolescents, harmful content was distressing and elicited negative emotional reactions, especially if the encounter happened while they were seeking social support or positive content related to their difficulties (Weinstein et al., 2021). However, some adolescents described how accidentally encountering distressing content could also be helpful, as it gave them a sense of shared experience (Radovic et al., 2017).

Some adolescents seek harmful content intentionally, for example, to find new ways of concealing injuries or to learn new methods of self‐harm. This is corroborated by quantitative findings showing that adolescents who received support for their mental health difficulties from in‐ or outpatient services were more likely to access harmful online content than their healthy peers (Mullen et al., 2018). Among adolescents receiving inpatient help, females and those who did not identify as either male or female were more likely to view or share online content related to self‐injury than males (Nesi et al., 2019; Nesi, Burke, Lawrence, et al., 2021). Online imagery seems to play a particularly important role in adolescent self‐harm practices: it could be a part of the ritual, encourage more severe injuries or trigger the felt need to self‐harm (Jacob et al., 2017). Ultimately, both intentional and unintentional access to harmful content could exacerbate adolescents' difficulties or hamper their recovery.

Some adolescents described using social media to create and share their content as part of self‐expression and coping with mental health difficulties. This could be achieved either through sharing images of their wounds (Jacob et al., 2017), posting suicidal thoughts or talking about their emotional state (Radovic et al., 2017; Weinstein et al., 2021). These qualitative accounts were supported by quantitative findings that showed adolescents with a diagnosis of depression tend to share their negative feelings online in a more intense manner than their nondepressed peers (Akkin Gurbuz et al., 2017). Such behaviour brought temporary relief and solicited attention and support from peers (Radovic et al., 2017). However, young people also acknowledged they often acted on impulse, said things online they did not mean or shared things they should have kept to themselves (Weinstein et al., 2021). In turn, such behaviour could create further vulnerabilities such as being detrimental to their friendships, leading to unwanted attention or even cyberbullying (Radovic et al., 2017; Weinstein et al., 2021).

### Cyberbullying

Cyberbullying refers to online behaviours whereby harmful or aggressive content about another person is shared via digital means (e.g. social media, applications, messages) (Livingstone, Stoilova, & Kelly, 2016). Adolescents who took part in two qualitative studies focused on describing how certain affordances of digital media facilitate and exacerbate this form of bullying (Radovic et al., 2017; Weinstein et al., 2021). These include the ease with which online bullies can remain anonymous, the public nature of social media and the ease of sharing any content. In combination, these enable online information to spread fast and reach a large audience, leading to more distress for the victim. Further, cyberbullying has no physical boundaries, so can easily spread from one context to another (e.g. from school to home). Finally, it is not just the anonymity that makes it harder to report online bullies but also the short‐lived nature of posts made through certain applications (e.g. Snapchat), which poses a challenge for adolescents to evidence cyberbullying.

Adolescents admitted to an inpatient unit with digital‐media‐related difficulties were more likely to be cyberbullied than those admitted for other reasons (Gansner, Belfort, Leahy, Mirda, & Carson, 2019). Cyberbullying, such as being a target of online rumours, was also significantly positively associated with self‐harm (OR = 15.51, 95% CI = 2.36, 101.80) and attempted suicide (OR = 16.89, 95% CI = 2.46–115.98) in a sample of adolescents receiving treatment in an inpatient service (Lanzillo et al., 2021). Compared with healthy peers, adolescents diagnosed with depression reported both significantly higher scores on a measure of cybervictimisation and also higher scores on a questionnaire assessing their behaviour as cyberbullies (Ucar et al., 2020).

In sum, the qualitative studies reviewed illustrated adolescents' awareness of how the affordances of the digital world may both facilitate and change the nature of bullying. Moreover, quantitative findings suggest that adolescents with mental health conditions may be more likely than their nonvulnerable peers to be both victims and perpetrators of cyberbullying. However, there are too few studies on this theme included in the review to draw a robust conclusion about cyberbullying.

### Self‐regulation of digital engagement

Three of the five qualitative studies explored issues related to self‐regulation during digital engagement (Hadwiger et al., 2019; Radovic et al., 2017; Weinstein et al., 2021). Despite including different clinical groups and a somewhat different focus in each of these studies, their findings were very similar. That is, young people reported substantial difficulties in using digital media in moderation or abstaining from using digital media. They described feelings such as ‘craving’ social media and being ‘addicted’. Some participants expressed the view that these feelings were reinforced by the constant availability of new online content and social feedback (Radovic et al., 2017; Weinstein et al., 2021). Others felt the pressure to be always online to be able to respond to their peers or offer prompt support, which then prevented them from taking a break from social media (Weinstein et al., 2021). In the case report published by Hadwiger et al. (2019), two male adolescents were so immersed in their digital activities (i.e. gaming) that they were forgetting to eat.

Difficulties with regulating digital media use were also reported in quantitative studies. Three studies (Alpaslan et al., 2016; Onat et al., 2019; Ucar et al., 2020) used the Young Internet Addiction Test (YIAT; Young, 2009) to compare symptoms of internet addiction in adolescents with a diagnosis of major depressive disorder and healthy controls. All studies reported significantly higher YIAT scores in the clinical groups suggesting overall more problematic internet use. Similarly, adolescent patients referred for internalising disorders reported significantly more difficulties with regulating use during the COVID‐19 lockdown than those referred for other problems (Werling et al., 2022). Finally, problematic internet use was significantly positively correlated with symptoms related to self‐injury (*r* = .203, *p* < .001), affective disorders (*r* = .300, *p* < .001), anxiety (*r* = .220, *p* < .001) (Meszaros et al., 2020) and depression (OR = 1.19, 95% CI = 1.16 1.21) (Li et al., 2021). In contrast, a study conducted by Onat et al. (2019) did not find a significant association between scores on the YIAT and self‐reported symptoms of depression (*r* = .001, *p* = .989). While the latter study did include a clinically depressed group, correlations were reported for the combined clinical and control groups, which might explain the difference in the results between the studies.

Three further studies examined the association between symptoms of depression and internet addiction using different measures (Firat et al., 2018; Martin‐Fernandez et al., 2016; Sahin & Usta, 2020). In a study conducted by Sahin and Usta (2020), adolescents diagnosed with major depressive disorder recruited from outpatient services completed the Social Media Use Disorder Scale (SMDS). They also measured smartphone ‘addiction’. There was an overall significant positive correlation between symptoms of depression and the SMDS (*r* = .301, *p* < .05) but no correlation between depression symptoms and smartphone ‘addiction’ (*r* = .242, *p* > .05). Further, Firat et al. (2018) reported a significant positive correlation between problematic mobile phone use and symptoms of depression and anxiety (*r* = .511, *p* < .001 and *r* = .487, *p* < .001, respectively) in a group of adolescents receiving inpatient treatment. Finally, 46% of the sample of adolescents receiving help for internet or video game problems had a comorbid internalising disorder (i.e. affective disorder, anxiety disorder or cluster C personality disorder). When the internet was not available to them, 59% of these adolescents reported feeling helpless and 26% reported feeling bored (Martin‐Fernandez et al., 2016).

In a series of studies, Gansner and colleagues examined problematic internet use in adolescents receiving help from both inpatient and outpatient mental health services. First, although adolescents with digital media‐related inpatient admissions did not report higher symptoms of depression, they felt more hopeless and were more likely to plan for suicide before admission compared with adolescents admitted for other reasons (Gansner, Belfort, Leahy, et al., 2019). Second, outpatient participants with a diagnosis of anxiety reported less problematic use than those with no such diagnosis. There was also no significant difference between the groups with and without a depression diagnosis. However, there was a significant positive correlation between problematic use and a self‐reported measure of anxiety symptoms (*p* < .05), while the positive correlation between problematic use and self‐reported depression symptoms approached significance (*p* = .05) (Gansner et al., 2020). A second study found that adolescents with problematic use scores above the clinically significant threshold were significantly younger and more likely to have clinically elevated depression and anxiety symptoms scores (Gansner, Nisenson, Lin, Pong, et al., 2022).

To deepen the understanding of problematic internet use and symptoms of depression and anxiety in adolescents receiving help in an outpatient clinic, Gansner, Nisenson, Lin, Carson, and Torous (2022) measured problematic use and recorded the number of phone sessions and phone checks (i.e., using the phone for less than 15 s at a time). Problematic internet use and self‐reported depression symptoms were negatively correlated with the number of daily phone checks and phone sessions. The authors suggested that adolescents with mental health conditions might engage with digital media as a way of escape or distraction from acutely worsening depressive symptoms. On the days when these adolescents are less able to access their smartphones, symptoms of problematic internet use appeared to be more prominent. There were no significant associations between phone sessions and phone checks and symptoms of anxiety.

In summary, the finding that adolescents with a diagnosis of depression have difficulties regulating their internet use is largely consistent across studies. There is also a relationship between self‐reported symptoms of anxiety, depression, self‐injurious behaviour and problems with self‐regulation of digital engagement. Difficulties with accessing digital devices at the time when adolescents experience more severe mental health difficulties may exacerbate ‘cravings’ and feelings of ‘addiction’ related to digital engagement.

## Discussion

This review aimed to compare and contrast the digital experiences of adolescents with clinically significant anxiety, depression, eating disorders or nonsuicidal self‐injury and to establish whether these young people are at greater risk of digitally related harm compared with nonclinical groups. The review also aimed to identify the potential benefits of digital experiences for adolescents with these conditions and to answer the question of whether studies of digital experiences of adolescents with emotional conditions can inform clinical practice.

A variety of methodological approaches and measures of digital experiences in the reviewed studies reflect the complexity of the topic. It remains challenging for both research and clinical practice that the digital world affords a wide variety of contents and interactions that can have diverse and contrasting impacts on mental health, such that for a host of contextual reasons, adolescents vary in their response to similar contents or interactions. Many of the included studies adopted cross‐sectional correlation designs. Although these have helped characterise the relationships between adolescent digital experiences and mental health outcomes, they do not allow us to draw causal inferences or establish a temporal sequence of events. To determine when the association developed, or how it has changed over time, we need longitudinal or experimental research designs. We should also note the clinical heterogeneity of the participants in the included studies, in terms of the type of problem they experienced, the severity of symptoms and the clinical setting from which they were recruited. On the one hand, this is a real strength of this review as its findings can be applied more broadly. On the other hand, such breadth inevitably leads to somewhat reduced focus and precludes us from making more nuanced inferences about the specific clinical groups.

First, many digital practices, for example, connecting with peers or seeking distraction, appear universal across the different conditions. One disorder that stands out as distinct is self‐harm. It appears that online imagery plays an important role in self‐harm rituals and this group may seek such harmful content intentionally (Jacob et al., 2017), while other groups may stumble upon it when seeking positive content (Radovic et al., 2017). Due to the small number of studies, this is a tentative finding that needs further investigation.

Second, we were not able to answer the question of whether digital experiences are different for adolescents with anxiety, depression, eating disorders or nonsuicidal self‐injury. The literature is too limited to identify robust themes that describe experiences unique to each disorder. There is a particularly striking lack of studies including samples with clinically diagnosed eating problems. Our searches identified only one such study (Hadwiger et al., 2019). Moreover, there have been too few direct comparisons between the conditions and indirect comparisons are not possible because of the heterogeneity in the concepts and measures used in the studies of different clinical groups. Finally, many studies included adolescents with multiple mental health disorders diagnoses, which substantially reduced the opportunity to identify mental health disorder‐specific digital experiences. Therefore, future research should include groups with clearly distinct mental health conditions to ensure results of clinical benefit. Moreover, future research should use designs that include both risk and opportunity outcomes so that we can weigh the relative risk and opportunity outcomes within a single study rather than trying to draw conclusions across studies.

Third, adolescents with clinical‐level mental health difficulties may be more vulnerable to digitally mediated harm compared with peers, who do not have mental health difficulties. A large proportion of the included studies focused on ‘problematic use’, an umbrella term for a variety of maladaptive digital practices including loss of control over use and internet/smartphone addiction. One consistent finding is that young people diagnosed with depression report more problematic use and difficulties in regulating their digital engagement than their nonclinical peers (Alpaslan et al., 2016; Onat et al., 2019; Ucar et al., 2020). This pattern of difficulties was also observed when adolescents with general internalising problems were compared with other clinical groups (Werling et al., 2022). Moreover, when clinically vulnerable adolescents experience an episode of severe mental health decline, being unable to access digital devices at the time may exacerbate ‘cravings’ (Weinstein et al., 2021). The studies that focused on problematic use are an important first step in developing a better understanding of what kind of effects the digital world may have on adolescent mental health. However, problematic use, whether it relates to the internet, social media or smartphones, should not be treated as undifferentiated activity and only through research using robust and nuanced measures of digital engagement we can advance our understanding.

Fourth, the reviewed literature suggests that digital media may provide adolescents who have mental health difficulties with positive social experiences, instant support from peers and mental health professionals and social acceptance (Nesi et al., 2019; Radovic et al., 2017; van Rensburg et al., 2016; Weinstein et al., 2021). However, male sex and greater severity of depression symptoms may reduce opportunities for benefiting from these positive digital practices (Akkin Gurbuz et al., 2017; Nesi et al., 2019). Some social connections could also exacerbate psychological difficulties and lead to the normalisation of pathological behaviour. In addition to interacting socially online, solitary digital practices (e.g., viewing content without engaging with others) may help some adolescents to find distraction and temporary relief from their psychological difficulties. However, such a way of coping may be maladaptive and could be linked to difficulties in regulating use. The algorithmic nature of the digital environment, with its ability to continuously personalise, curate and suggest even more extreme media content, plays an important role in adolescents' use regulation difficulties (Stoilova et al., 2021) but we found that this has been understudied with clinical populations. This is a critical aspect that needs further investigation and would have significant implications for clinical practice in terms of reversal.

Fifth, young people embrace the new opportunities for communication, networking and boosting social popularity afforded by the digital world. However, this may lead to two kinds of negative outcomes for adolescents who have emotional difficulties. One, it may motivate engaging in risky or problematic online practices to gain positive social feedback or to appear popular (Jacob et al., 2017; Radovic et al., 2017). Two, viewing heavily filtered posts that depict only happy and successful events and that are a poor reflection of the more mundane reality can evoke feelings of missing out and lead to negative social comparisons that lower the mood of clinically vulnerable adolescents (Weinstein et al., 2021).

Finally, our database searches did not identify any clinical recommendations or guidelines that fell within the specific inclusion criteria of this review. However, we note that the more general position statements that concern digital technology and child and adolescent physical and mental health have been issued by the European Academy of Paediatrics (Hadjipanayis et al., 2019), the American Academy of Paediatrics (Reid Chassiakos et al., 2016) and by the Royal College of Psychiatrists (Dubicka & Theodosiou, 2020). These recommend that clinicians should actively familiarise themselves with the kinds of digital and social media platforms that children and adolescents use and be aware of the positive and negative impacts these might have on children's and adolescents' lives. Clinicians should also discuss digital experiences during clinical contact and make evidence‐based recommendations regarding technology use. It is vital that questions about children's and adolescents' digital experiences are included in psychosocial assessment and psychiatric formulation. Not asking these questions risks missing an important part of the picture, both in terms of factors that maintain or escalate problems and those that could mitigate them.

## Conclusions

This review provides evidence that many online experiences (both positive and negative) are shared by adolescents with different mental health difficulties. The digital world provides adolescents with opportunities for social connection, and support and allows distraction from their psychological problems. At the same time, some young people may find it difficult to self‐regulate how they engage with the digital world and their digital experiences can exacerbate mental health difficulties or lead to the normalisation of pathological behaviour. Future research should directly compare the digital experiences of individuals with different pre‐existing mental health conditions and be designed in a way to better understand the more nuanced impact of digital experiences on the specific clinical groups.

## Ethical information

This is a review of the published studies, for which ethical approval is not required.

## Supporting information


**Table S1.** Review data extraction table.

**Appendix S1.** Search Terms.
