# Supplementary material for: Review: Digital experiences and their impact on the lives of adolescents with pre‐existing anxiety, depression, eating and nonsuicidal self‐injury conditions – a systematic review
Source: Child Adolesc Ment Health. 2022 Dec 7;28(1):22–32. doi: 10.1111/camh.12619 (PMC10108198; doi:10.1111/camh.12619)
Supplement: Supplementary file 1 — Table S1. Review data extraction table. [file CAMH-28-22-s001.docx]

Table S1. Review data extraction table.

| Authors (year) | Study type | Aims | Age | Sample size | Sex (% male) | Setting | Country | MH condition | MH measure | Digital engagement | Analysis | Findings | WoE D |
| --- | --- | --- | --- | --- | --- | --- | --- | --- | --- | --- | --- | --- | --- |
| Akkin Gurbuz, H. G., Demir, T., Gokalp Ozcan, B., Kadak, M. T. and Poyraz, B. C. (2017) | Case control | Evaluate the incidence of depressive disclosure on social media habits among depressed and non-depressed adolescents. | Range: 13-18; Depression group: 15.29 (1.32), Control group 15.23 (1.25) | N = 108; Depression group = 53, Control group = 55 | Depression group, 38%; Control group 42% | Outpatient | Turkey | Depression | K-SADS-PL | Type of social media used, time online and on social media, meeting strangers, online opportunities (learning), online depression disclosure. | Quantitative | Non-depressed adolescents used the Internet to do homework significantly more frequently than depressed peers (χ2 = 9.532, p = .002). Depressed boys did significantly less homework online than girls (χ2 = 8.094, p = .004). No statistical difference between depressed and control individuals in meeting in-person with people they met online. Depressed adolescents spent statistically more time online and on SNS. There are no differences in the types of social media used. Most participants found it easier to express themselves on SNSs than offline at least once in a while with no differences between the groups (Mann–Whitney U = 1422.000, p = .219). No significant differences were found in disclosures of depressed mood on SMS. Depressed group share their negative feelings on SNSs more intensely than controls. Irritability (U = 1587.500, p = .001), suicidal ideation U = 1578.000, p = .011), feelings of guilt and worthlessness (U = 1574.000, p = .005), concentration loss (U = 1493.000, p = .027), and anhedonia (U = 1536.500, p = .029) were all disclosed significantly more on SMS by the depressed group. No somatic symptoms of depression (lethargy U = 1169.000, p= .533, loss of appetite U = 1357.500, p = .187, insomnia U = 1389.000, p = .240, and psychomotor activity U = 1359.000, p = .345), were disclosed significantly more on SMS by the depressed group. | 3.0 |
| Alpaslan, A. H., Soylu, N., Kocak, U. and Guzel, H. I. (2016) | Case control | To study the relationship between PIU and MDD. | Range: 12-18;  MMD: Mean = 15.22 (1.57), Control group: Mean = 15.05 (1.61) | N = 220;  MDD = 120, Control group = 100 | MDD: 37.5%; Control group: 42% | Outpatient | Turkey | Depression | CDI | YIAT | Quantitative | PIU was significantly more common for depressed group than controls (χ2 = 27.26, p < 0.001) after controlling for age and gender. No correlation between suicidal ideation or attempts and PIU amongst depressed adolescents. YIAT scores significantly higher in depressed group than controls after controlling for age and gender (np2 = 0.358, p < 0.001). No difference in PIU between those who had and those who had not attempted suicide in the last 6 months (χ2 = 2.35, p = 0.188). Hopelessness CDI scores were much higher in depressed participants with PIU than depressed individuals without PIU (z = 3.10, p = .002). | 2.7 |
| Cao, J., Truong, A. L., Banu, S., Shah, A. A., Sabharwal, A. and Moukaddam, N. (2020) | Crossectional correlation | To investigate if phone apps are useful in evaluating and monitoring depression symptoms in depressed adolescents. | Range = 12-17; Mean = 14.93 (1.59) | N = 13 | 15% | Outpatient | USA | Depression | HAM-D. HAM-A. PHQ-9. | Call information: timestamp, phone number, type (incoming, outgoing, and missing), and duration. Text information: timestamp, phone number, type (incoming or, outgoing), and number of characters. Percentage of time the screen was on for each hour. | Quantitative | Higher depression score is significantly correlated with lower social interaction level; shorter phone call duration -0.60 r < -0.40 and fewer text messages -0.60 < r < -0.40 (both p > .05), but not with screen time. No significant relationship between screen usage or ambient light intensity and HAM-D, HAM-A, and PHQ-9. | 3.0 |
| Firat, S., Gul, H., Sertcelik, M., Gul, A., Gurel, Y. and Kilic, B. G. (2018) | Crossectional correlation | To study the relationship between sociodemographic characteristics, psychiatric symptoms and emotion regulation problems | Range = 12 -18; Mean = 15.28 (1.65) | N = 150 | 41% | Inpatient | Turkey | Depression | BSI shortened. DERS. | PMPUS | Quantitative | Problematic smartphone usage was negatively correlated with academic achievement (p=.001 Cramer's V=0.461), and positively to disciplinary school punishment (p=.001, V = 0.290). Problematic phone use was positively associated with depression (Z = −6.427, p < .0001), anxiety (Z = −6.303, p < .0001), phobic anxiety (Z = −6.018, p <. 0001), somatisation (Z = −7.24, p < .0001), obsessive-compulsive (Z = −5.122, p < .0001), interpersonal sensitivity (Z = −5.931, p < .0001), hostility (Z = −5.888, p < .0001), paranoid ideation (Z = −6.173, p < .0001), psychoticism (Z = −6.275, p < .0001), emotional clarity (Z = −2.730, p = .006), limited emotional regulation strategies (Z = −5.340, p < .0001), difficulty engaging in goal directed behaviour (Z = −3.631 p < .0001), and impulsivity (Z = −4.799, p < .0001) after controlling for age. Of DERS, only impulsivity significantly predicted problematic smartphone use (β = 0.482, p < .0001). Of BSI symptoms of depression, only somatization (β = .577, p < .001), interpersonal sensitivity (β = - .458, p = .007), and hostility (β = .461, p = .003) significantly predicted problematic smart phone use. | 2.0 |
| Ganser, M., Belfort, E., Leahy, C., Mirda, D. and Carson, N. (2019) | Crossectional correlation | To study the digital media use in psychiatrically hospitalized adolescents | Range = 12-20; Mean = 15.6 | N = 68 | 38% | Outpatient | USA | Multiple | PHQ-2 | Frequency, risk (e.g., cyberbullying, sexting), parental monitoring and PIU (via the MIDI, assessing presence of impulse control disorder, mood disorder, anxiety disorder, and psychosis). | Quantitative | Those with digital media-related admissions spend more time in virtual worlds (χ2 = .48, p = .490) and on SMS (χ2 = 8.12. p = .004) than those not admitted. Similarly, they are more likely to experience cyberbullying (χ2 = 8.54, p = .003), and sexting (χ2 = 5.62, p = .020), but not PIU (χ2 = 2.04, p = .15). They did not have a higher risk of recent depression (χ2 = 1.39, p = .230) or self-harm (χ2 = 2.41, p = .120), but had a significantly higher risk of hopelessness (χ2 = 3.94, p =.040) and suicide planning (χ2 = 6.79, p = .009). | 2.0 |
| Gansner, M., Nisenson, M., Carson, N. and Torous, J. (2020) | Longitudinal correlation | To study the feasibility of using app-based EMA to identify and characterize the relationship between mood symptoms and PIU in adolescents during mental health treatment. | Range = 12-23; Mean = 15.5 | N = 25 | 32% | Outpatient | USA | Multiple | PHQ-8; GAD-7 | PIU-SF-6 | Quantitative | The majority of participants reported an increased awareness of the relationship between their mood symptoms and digital media use (83.3%). A third talked more about their digital media use with their mental health providers. participants with anxiety disorders were more likely to report increased attunement to their psychiatric symptoms in relation to their digital media (p < .05). Youth with anxiety disorders used the app more frequently to report their mood (t = −2.77, p < 0.05). There appeared to be no correlation between PIU severity and a participant's age or gender. The severity of PIU was not associated with depression symptoms (adjusted R2 = 0.12, intercept = 1.46, slope = 0.39, p = .050) but it was associated with symptoms of anxiety (adjusted R2 = 0.16, intercept = 1.40, slope = 0.46, p = .032). | 2.0 |
| Gansner, M., Nisenson, M., Lin, V., Carson, N. and Torous, J. (•2022) | Longitudinal correlation | To assess the relationships between PIU, mood symptoms, and daily smartphone engagement | Range = 12-23; Mean = 15.6 | N = 28 | 50% | Outpatient | USA | Multiple | PHQ-8.  GAD-7: | PIU-SF-6 | Quantitative | 5.57 hours of smartphone usage daily on average. 68.7 phone sessions during that time, with each session averaging 7.79 minute. The average daily number of phone checks was 29.7. Total screen time and session duration were unrelated to scale score severity, both PIU-SF-6 and PHQ-8 scores were less severe with an increasing number of daily phone checks (p = .03, p = .01, respectively) and phone sessions frequency (p = .046, p = .02, respectively). Depression symptoms were worse on days with decreased smartphone engagement. On days when these youth are less able to access their smartphones, symptoms of PIU were more noticeable. PIU was negatively associated with departure from daily routines. There was no significant relationship between screen time and PIU. | 2.0 |
| Gansner, M., Nisenson, M., Lin, V., Pong, S., Torous, J. and Carson, N. (2022) | Crossectional correlation | Aims to examine potential pandemic-associated changes in digital media use in outpatient mental health treatment. | Range = 12-23; Mean = 15.30 (2.74) | N = 69 | 48% | Outpatient | USA | Multiple | PHQ-8;  GAD-7. | PIU-SF-6: Digital media use (based on Apple’s daily screen time reports). | Quantitative | PIU is positively associated with experiencing clinically significant symptoms of anxiety and depression. SMS used more during the pandemic (p = .049) and especially those who had COVID (p = .01). COVID cohort more likely to meet criteria for PIU (p = .02). SMS most popular type of app used during the pandemic. PIU-SF-6 scores of ≥15 significantly younger and more likely to have clinically elevated PHQ-8 and GAD-7 scores (p = .003). | 2.0 |
| Hadwiger, A. N., Middleman, A. B. and Pitt, P. D. (2019) | Case observation | To explore the potential association between ARFID and gaming behaviour. | Range = 15-17; Mean = 16 | N = 2 | 100% | Observations taken as inpatient and outpatient | USA | Multiple | Mean Estimated Body Mass Index (MEBMI). | Interview to assess presence of IGD was being considered as a possible diagnosis by the medical community but was not officially recognized as a diagnosable condition. | Qualitative | A comorbid relationship of two adolescent risk behaviours - restrictive eating/purging and excessive gaming was observed - both of which can result in negative medical and mental health outcomes. Both patients had restrictive eating behaviours and excessive gaming - and these behaviours created significant functional impairment (leading to medical and psychosocial compromise) and prolonged hospital stays that potentially further interfered with their development. Both patients also had an anxious disposition. | 1.7 |
| Jacob, N., Evans, R. and Scourfield, J. (2017) | Qualitative study | To explore how young people understand and use online images of self-harm. | Range = 16-24; Mean = 19 | N = 21 | 14% | Community | UK | Self-harm | Semi-structured interviews exploring NSSI, support systems used (either sought professional help for self-harm, or present to Accident and Emergency Department for injuries), use of internet. | Semi-structured interviews exploring internet use as part of self-harming practice, influence of online imagery, appeal of SMS for young people engaging in NSSI. | Qualitative | The majority of participants engaged with online spaces to support and further develop a pre-existing set of self-harming practices. Engagement with online communities often led to an exacerbation of self-harm due to normalisation and increased exposure and access to new techniques. Participant's used digital stores to get around barriers to prevent self-harm - such as buying blades and other age restricted items online. | 2.7 |
| Lanzillo, E. C., Zhang, I., Jobes, D. A. and Brausch, A. M. (2021) | Crossectional correlation | To investigate the association between cyberbullying and self-injurious thoughts and behaviours separately; to examine how being the subject of online rumours, illicit photographs, and threatening messages related to NSSI, SI, and SA history in a psychiatric adolescent. | Range = 12-17; Mean = 14.3 (1.44) | N = 64 | 31% | Inpatient | USA | Self-harm | Suicide Status Form (used in CAMS). Self-Harm Behavior Questionnaire. | Victim of cyberbullying (reports of whether they have been the subject of online rumour, receiving online threats, illicit photo shared online). | Quantitative | 60% experienced cyberbullying or online rumour, 42% had experienced online threats, and 10% had been in an illicit photo. Reported types of cyberbullying, such as online rumours (OR = 15.51 95% CI = 2.36–101.80, p = .004), but not threatening messages were associated with NSSI. Specifically, victims of online rumours were 15x more likely to engage in NSSI; and all participants who reported being in illicit photographs reported NSSI; participants who endorsed involvement in an online rumour were nearly 17 times more likely to report SA. | 3.0 |
| Li, Z. L., Liu, R., He, F., Li, S. Y., Zhao, Y. J., Zhang, W. Y., Zhang, Y., Cheung, T., Jackson, T., Tang, Y. L. and Xiang, Y. T. (2021) | Crossectional correlation | To examine the prevalence of IAD and its correlates among clinically stable adolescents with psychiatric disorders | Range = 10-17;  Mean = 14.73 (1.94); Non-IAD group  Mean = 14.76 (2.00), IAD group14.65 (1.78) | N = 1454;  Non-IAD = 1,000;  IAD = 454 | 38.80% | Outpatient | China | Depression | PHQ-9. Information about psychiatric diagnosis, perceived academic pressure, relationship with parents, COVID-19 concern, daily physical exercise, media use, difficulty seeing psychiatrists, treatment adherence, and illness relapse. | IAT. | Quantitative | Gender (χ2 = 28.67, p < .001), residence (χ2 = 5.44, p < .02), principal psychiatric diagnosis (χ2 = 30.56, p < .001), PHQ-9 scores (χ2 = -22.12, p < .001), perceived academic pressure (χ2 = 26.08, p < .001), relationship with parents (χ2 = 77.70, p < .001), COVID-19 concern (χ2 = 22.17, p < .001), exercise (χ2 = 22.76, p < .001), difficulty seeing psychiatrists (χ2 = 9.40, p = .009), treatment adherence (χ2 = 39.35, p < .001) and illness relapse (χ2 = 39.35, p < .001) significantly differed between adolescent patients with IAD and those without IAD. Poor relationship with parents (OR = 2.34, 95%CI: 1.49–3.68, p < .001) and higher PHQ-9 total scores (OR = 1.19, 95%CI: 1.16–1.21, p < .001) were predictors of higher risk of IAD, while exercise durations (OR = 0.67, 95%CI: 0.46–0.98, p = .04) and rural residence ( OR = 0.62, 95%CI: 0.46–0.85, p = .003) were associated with significantly lower risk of IAD. | 2.0 |
| Martin-Fernandez, M., Matali, J. L., Garcia-Sanchez, S., Pardo, M., Lleras, M. and Castellano-Tejedor, C. (2016) | Transveral | To describe profiles of adolescents with IGD. | Range = 12-17; Mean = 14.8 (1.45); Internalizing mean = 15.19 (1.62); Externalizing mean = 14.48 (1.23) | N = 59; Internalizing, N = 27, Externalizing, N = 31 | 96.60% | Outpatient | Spain | Multiple | K-SADS-PL: | Online videogame data were recorded: When games were played, time preference for gaming, types of applications used (online games, MMORPG and/or chats), to assess how patients felt when they were taken away (bored or helpless). | Quantitative | 80.6% of the externalizing group stated that video games were played mostly for leisure purposes, while 68.7% of internalizing patients claimed that they played to hide themselves away or avoid discomfort. When games are taken away, 59.3% of the internalizing group reported feeling incapable of dealing with situation, while 61.3% of externalizers reported boredom. 74.1% of internalisers preferred night-time gaming, compared to 29% of externalisers. Externalisers expressed a greater variety of applications, online games (38.7%), MMORPG (25.8%), MMORPG with chat (29%) and online chat alone (6.5%). In contrast, the internalisers play mainly MMORPG (81.5%), MMORPG with chat (11.1), 3.7%, online chat alone, and offline games (3.7%). | 3.0 |
| Meszaros, G., Gyori, D., Horvath, L. O., Szentivanyi, D. and Balazs, J. (2020) | Case control | To determine whether there is a connection between PIU and NSSI which is mediated by internalising and externalising psychopathology. | Mean = 15.2 (1.31) | N = 363; Clinical, N = 202, Nonclinical, N = 161 | 49.30% | Psychiatric inpatients and community (nonclinical) | Hungary | Multiple | SDQ; DSHI; Kid: Interviews assessing the existence of various mental health disorders. | YDQ. | Quantitative | NSSI was significantly more frequent among those who showed threshold symptoms on SDQ than in the subthreshold group [H(3) = 53.293, p <.001]. Frequency of NSSI was significantly lower in ‘normal’ internet users than in ‘maladaptive’ and ‘pathological’ internet users [H(2) = 10.039, p <.05 p = .007]. The relationship between PIU and NSSI is fully mediated by all psychopathological factors (M.I.N.I. kid diagnoses) except for obsessive-compulsive disorder (OCD), alcohol abuse and dependence, and adjustment disorder. | 2.0 |
| Mullen, G., Dowling, C. and O'Reilly, G. (2018) | Case control | To understand internet use by young people experiencing mental health difficulties compared to controls. | Range = 12-19 Inpatient: R= 13-17; Outpatient: R=13-19; Community: R=12-18 | 299; Inpatient, N = 29, Outpatient, N = 33, Vulnerable community, N = 56, Healthy community, N = 181 | 36% | Four Groups: (1) attending inpatient services; (2) attending outpatient services; (3) a community group with mental health concerns and no clinical support; and (4) a regular community group. | Ireland | Multiple | Y-PSC. | Frequency of website use across entertainment, interest, information, gaming, harmful content, and porn. | Quantitative | Those in the inpatient and outpatient groups visited more potentially harmful websites compared to controls [ χ2 (3, 299) = 15.06, p = .002]. Those attending inpatient and outpatient services showed aspects of both more risky and less risky use. The vulnerable community group reporting no mental health difficulties showed least risky use. The group experiencing difficulties but not receiving support showed consistently high risky use, suggesting this is a particularly vulnerable group. The inpatient group used the internet less during the evenings/early night compared with the vulnerable and healthy community groups, [ χ2 (3, 299) = 11.92, p = .008]. Inpatients reported more use late at night, compared with all other groups, [ χ2(3, 299) = 12.43, p = .006]. Outpatients reported less use in the evening/ early night compared with the vulnerable and healthy community groups. Less of the vulnerable community group used a family laptop/computer [ χ2(3, 299) = 13.73, p = .003], and more reported use during school time [ χ2(3, 299) = 7.99, p = .046]. Weak correlations were observed whereby lower SEG participants used less Information Sites [p = 0.001, ρ = −0.19], males used more Online Gaming sites [p = 0.000, ρ = 0.23], males reported less use of Information Sites [p = 0.001, ρ = − 0.19], and the oldest group accessed more Potentially Harmful sites than younger groups [p = 0.001, ρ = 0.19]. | 2.3 |
| Nesi, J., Burke, T. A., Extein, J., Kudinova, A. Y., Fox, K. A., Hunt, J. and Wolff, J. C. (2021) | Crossectional correlation | To examine positive and negative SMS experiences of psychiatrically hospitalized adolescents and explore differences in SMS use based on diagnostic presentation. | Range = 11-18; Mean = 15.34 (1.66) | 243 | 35% | Inpatient | USA | Diagnosis not specific - sample derived from patients who were hospitalized in a psychiatric inpatient facility. | SIQ-Jr: 7-point Likert scale assessing internalising symptomology and attention problems. | 8-point Likert scales assessing positive and negative SMS experiences, SMS use duration, Frequency checking SMS, Perceived overuse of SMS, Importance of SMS, and Emotional Responses to SMS Experiences. | Quantitative | SMS usage was not significantly associated with sleep disturbance (a path; b = - 0.19, se = 0.15; p = .229). There was no significant direct or indirect association between time on SMS and suicidal ideation [ direct b = 0.03, se = 0.03, p = .317; indirect effect b = - 0.01, se = 0.01, 95% CI ( - 0.04, 0.01) ], internalizing symptoms [ direct effect b = 0.05, se = 0.05, p = .315; indirect effect b = - 0.03, se = 0.02, 95% CI ( - 0.07, 0.02) ]; attention problems [direct effect b = 0.00, se = 0.04, p = .942; indirect effect b = - 0.02, se = 0.02, 95% CI (-0.05, 0.01)]. No significant associations were revealed between sleep disturbance and average duration of SMS use [r = -.02], frequency of checking [r = -.07], perceived importance [r = -.02], perceived overuse [ r = -.00], and positive emotional responses to SMS [r = -.11]. A significant association between sleep disturbance and negative emotional responses to SMS [r = .24, p < .01], suicidal ideation [r = .40, p <.01], internalizing symptoms [ r = .46, p < .01], and attention problems [ r = .40, p < .01]. | 2.0 |
| Nesi, J., Burke, T. A., Lawrence, H. R., MacPherson, H. A., Spirito, A. and Wolff, J. C. (2021) | Crossectional correlation | To explore patterns of online self-injury activities in adolescents at an in-patient setting due to risk of harm to self or others. | Range = 11–18, Mean = 14.88 (1.83) | N = 589 | 35.10% | Inpatient | USA | Multiple | SIQ-Jr. SA/NSSI: | Online NSSI activities: Likert scales assessing viewing NSSI content, sharing NSSI content, using technology to speak to others offline about NSSI, and using technology to speak to others online about NSSI, use of websites / apps used for NSSI activities. Function of online NSSI activities: Negative affect regulation, Boredom reduction, Positive affect enhancement, Reduced isolation, Self-expression, Recovery, Identity exploration. Consequences of online self-injury: Normalization of NSSI, thwarted recovery, behavioural triggering, social comparison, discovery of new NSSI methods. | Quantitative | 74.8% used SNS. 33.9% used text messaging or messaging apps. 18.1% used video sharing platforms, and 9.8% used websites specifically for people who self-injure. Class 1 (33.2%) reported moderate to low engagement in online NSSI activity for affect regulation and boredom reduction. They also reported below average identification with other reasons for NSSI online activities - specifically isolation reduction, self-expression, recovery and identity exploration. Class 2 (50.2%) reported average engagement in online NSSI activities for affect regulation and boredom reduction. They also reported above average endorsement of isolation reduction, self-expression, recovery and identity exploration. Class 3 (16.6%) expressed above average endorsement for online NSSI activities for affect regulation, boredom reduction, isolation reduction, self-expression, recovery, and identity exploration. | 2.3 |
| Nesi, J., Wolff, J. C. and Hunt, J. (2019) | Crossectional correlation | To examine SMS experiences of in-patient adolescents. To determine the prevalence of positive and negative SMS experiences in this population, and to explore differences in SMS use based on diagnostic presentation. | Range = 11-18; Mean = 14.6 | N = 433 | 38.40% | Inpatient | USA | Multiple | Lifetime history of a suicide attempt: measured with a single item from the Self-Injurious Thoughts and Behaviors Interview. | Binary choice questioning of positive and negative SMS experiences. | Quantitative | The five most popular SMS were Snapchat (76.4%) then text messaging (75.1); Instagram (71.8%); Facebook and Twitter (29.8%). Girls had significantly more positive and negative experiences on social media than boys. Compared to boys, girls reported viewing more content encouraging NSSI [χ2 = 6.50, p = .011], were cyberbullied more [χ2 = 11.98, p = .001] and talked to strangers more [χ2= 4.44, p = .035]. There was no difference between the sexes in terms of getting into a fight or argument online. 65.4% of all participants used SMS to distract from difficult situations and 57% to receive support or encouragement from friends, 37.4% compared themselves negatively to others on SMS, 30.7% felt left out or excluded, 14.8% had viewed content encouraging NSSI. Those with internalizing diagnoses were more likely compare themselves negatively to others [χ2 = 24.86, p < .001 for girls; χ2 = 7.00, p = .008 for boys], and having felt excluded [χ2 = 8.33, p = .004 for girls; χ2 = 9.94, p = .002 for boys]. | 2.7 |
| Onat, M., Ozyurt, G., Ozturk, Y. and Akay, A. P. (2019) | Crossectional correlation | To compare PIU between adolescents with MDD and controls. | Range = 12-18;  MDD mean = 14.70 (1.48), Control group mean = 15.02 (1.55). | N = 198; MDD, N = 97,  Control, N = 101 | MDD = 38.9%; Control = 42.3% | Outpatient | Turkey | Depression | K-SADS-PL; BDI. | YIAT. | Quantitative | The MDD group scored higher on the overall IAT score and on relationships, responsibilities and duration [all p < .001], but not mood [p > .05]. | 2.7 |
| Radovic, A., Gmelin, T., Stein, B. D. and Miller, E. (2017) | Qualitative study | To explore the main purpose for using SMS by adolescents with depression. To identify examples of positive and negative use, and to examine the relationship between negative use and mood. To explore how engagement in mental health treatment had resulted in changes in SMS use. | Range = 13-20; Mean = 16 (2.3) | N = 23 | 21.70% | Outpatient | USA | Depression | Currently receiving treatment for depression. | Semi-structured interview to assess SMS, positive and negative experiences using SMS, how use of different SMS sites varied, SMS and mood, oversharing, , stressed posting, triggering posts, and interactions with other depressed peers. | Qualitative | Most used SMS: Facebook [45%], Tumblr [18%], Twitter [18%], and Instagram [91%]. Positives uses include: To explore identity, experience independence, to gain social support, peer acceptance, and connection to others. Negative uses included: comparing themselves to others, cyberbullying, risky behaviour, posting negative content or using SMS for negative ways to cope with mood. PIU was defined as oversharing, stressed posting, and viewing triggering posts. | 2.7 |
| Sahin, B. and Usta, M. B. (2020) | Crossectional correlation | To investigate the relationship between SMS - and smartphone - use and severity of depression symptoms in adolescents. | Range = 13-18; Mean = 15.6 (1.2) | N = 56 | 30.10% | Outpatient | Turkey | Depression | K-SADS-PL; CDI. | SMDS;  SAS-SV. | Quantitative | SMS used: WhatsApp (100%), Instagram (88%), Twitter (64%), Snapchat (54%), Facebook (46%), and TikTok (46%). A significant positive correlation between depression score and social media use [r = .301, p < .05], but not between depression score and smartphone addiction [r = .242, p> .05]. Further, significant positive correlations between depression scores and these SMDS subscales preoccupation [r = .368, p < .05], tolerance [r = .394, p < .05], escape [r = .425, p < .05], deception [r = .397, p < .05], displacement [r = .409, p < .05], and conflict [r = .352, p < .05]. | 3.0 |
| Ucar, H. N., Cetin, F. H., Ersoy, S. A., Guler, H. A., Kilinc, K. and Turkoglu, S. (2020) | Case control | To compare cyberbullying, susceptibility to cyberbullying, cyber victimisation, and internet and digital game addiction in adolescents with and without depression. | Range = 12-17; Depression group mean = 14.6 (0.9); Control group = 14.4 (0.5) | N = 73; Depression group = 34, Control group = 39 | Depression group = 38.2%; Control group = 51.3% | Outpatient | Turkey | Depression | K-SADS-PL: RCADS-CV: | CBS;  CBSS; Cybervictimisation scale; IAS; DGAS. | Quantitative | Those with depression were more likely to use DMs and chat to engage on social media than controls [p = .001]. There was no difference in the numbers gaming, using online forums, email or doing online research [p > .05]. Significantly higher scores of cyberbullying, cyber victimization, and internet and digital game addictions in depressed participants compared to controls [all p < .05]. | 3.0 |
| Ucar, H. N., Eray, S., Kocael, O., Ucar, L., Kaymak, M., Lettieri, E. and Vural, A. P. (2018) | Crossectional correlation | To explore under which conditions - and how frequently - adolescents who have a diagnosed disorder share their symptoms on SNS. | Range = 12-18; Mean = 15.5 (1.7) | N = 224 | 41% | Outpatient | Turkey | Multiple | K-SADS-PL: |  | Quantitative | The three most frequently used SNS were: Facebook was used by 47.3% of the sample, Instagram by 23.2% and Snapchat by 6.7%. Mean (SD) internet time was 5.3h/day (4.3) and mean SNS use time was 2.8h/day (2.5). Three-quarters of participants share their feelings on SNS. Being female was negatively associated with symptom sharing [B = -.301, p < .01]. However, MDD and the amount of Internet and SNS use were positively associated with symptom sharing [MDD: B = .387, p < .01]; daily hours on Internet [B = .043, p < .01], and daily hours on SNS [B = .128, p < .001]. However, once the perceived value of SNS was introduced into the regression model, depression was no longer a significant predictor of the symptom sharing. | 3.0 |
| van Rensburg, S. H., Klingensmith, K., McLaughlin, P., Qayyum, Z. and van Schalkwyk, G. I. (2016) | Qualitative study | To explore how patients would like to incorporate SMS into communication with their psychiatric providers. | Range = 14-19; Mean = 16.1 | N = 20 | 25% | Outpatient | USA | Multiple | N/A | Participants’ experience of SMS and how they felt about communicating with health-care providers using social media. | Qualitative | Participants report that using SMS to access mental health professionals could allow for constant access to healthcare, meaning quick feedback from clinicians, access to support outside of scheduled appointments, and value of supportive presence on social media - but could cause anxiety if response time is slow. In addition. Participants also reported that using SMS to access mental health professionals could make it easier to open up, meaning less embarrassment than face-to-face interactions, communication resembling interactions with family and friends - but also concern that non-verbal communication would be lost and it would be easier to hide feelings. Finally, participants reported that they could be better monitored, meaning ease of clinicians becoming aware of safety concerns, clinicians may gain sense of longer-term changes in mental health - but there was also concern that content may be misinterpreted after being posted to SMS. | 2.3 |
| Weinstein, E., Kleiman, E. M., Franz, P. J., Joyce, V. W., Nash, C. C., Buonopane, R. J. and Nock, M. K. (2021) | Qualitative study | To explore negative and positive experiences related to social media/ smartphones use by suicidal adolescents. To explore how adolescents describe their disconnection from these technologies during inpatient hospitalization and views on a subsequent return to digital connectivity after discharge | Range = 13-18; Mean = 16.1 (1.6) | N = 30 | 30% | Inpatient | USA | Multiple | Currently hospitalized for a recent SA or SI. | Semi-structured interview: Type of social media accounts and frequency, uses (positive and negative) and views on both disconnecting during hospitalization and anticipated re-entry post-hospitalization. Interviews focused on previous uses when not hospitalized, current experiences disconnecting, and future expectations for following discharge. Questions considered SMS, smartphones, messaging, gaming, and other apps. | Qualitative | Actively used SMS: Instagram [ 60% ], Snapchat [ 76.7% ], and Facebook [ 16.7% ]. Nine aspects of SMS identified as risks & challenges: (1) Regulating use, feeling addicted [40%]. (2) Pressure of metrics (likes, follows) [37%]. (3) Access to ‘triggering,’ depressogenic, or NSSI content [33%]. (4) Cyberbullying, hostility, and direct exclusion [30%]. (5) Upward social comparisons (e.g., social lives, body-image) [30%]. (6) Friendship expectations, no alone time [30%]. (7) FOMO [27%]. (8) Having a perfect profile [23%]. (9) Expression of emotions harming relationships [10%].  Six aspects were identified as opportunities & benefits: (1) Social connection [67%]. (2) Access to positive content [60%]. (3) Seeking support from others [53%]. (4) Access to resources for mental health [37%]. (5) Shared interests [37%]. (6) Self-expression opportunities [17%]. 67% found disconnection during hospital stay positive. Some reported missing social connections, better sleep, focus, recovery, less pressure to post, and less feeling excluded. 10% found it negative - loss of coping content and social connection. Post-discharge reports excited to reconnecting friends, SMS integral to social re-entry, concern by the volume of content to “catch up” on: re-engaging with depressogenic content; what others said about them online; explaining absence; feeling addicted again. 60% expressed ambivalence or apathy, some described only positive [13%] negative [20%] feelings. | 2.7 |
| Werling, A. M., Walitza, S., Gerstenberg, M., Grunblatt, E. and Drechsler, R. (2022) | Case control | To compare the impact of  the spring 2020 lockdown on digital engagement and mental well-being in adolescents internalising individuals with adolescents with other psychopathology. | Range = 12-18; Internalising, mean = 15.7 (1.5), Non - internalising, mean = 15.2 (1.7). | N = 178; Internalising, N = 89; Non-internalising, N = 89 | Internalising = 15.7%; non-internalising = 25.8% | Outpatient | Switzerland | Multiple | Psychopathology, emotional and behavioural problems and worry were measured with the European collaboration study on the impact of COVID-19 on existing mental health problems (CRISIS). | PUI-SQ. | Quantitative | Leisure media use: both groups reported more frequent SMS use during the lockdown than before. Social media time: no difference between groups either before, during or after the lockdown [all p < .05]. ID group reported more SMS impact on mood [χ2 = 12.218, .007] [ p < .05 ] and reported more unsuccessful attempts to reduce media time than non-ID group [χ2 = 9.004, p = .029 ]. During lockdown, Non-ID group SMS time was positively associated with a worsening of symptoms, whereas in the ID group, SMS time was positively correlated with improved symptoms. In both groups, the impact of media use on everyday life strongly predicted emotional distress [internalizing, B = .553, p < .001; non-internalizing, B = .405, p < .001] and behavioural problems [internalizing, B = .493, p < .001; non-internalizing, B = .453, p < .001]. In ID group, emotional distress was additionally inversely related to gaming time [B = - .310, p < .001] and addictive tendencies [- .226, p = .36]. | 3.0 |

**ABBREVIATIONS:**

ARFID = Avoidant/Restrictive Food Intake Disorder; BDI = Beck Depression Inventory; BSI = Brief Symptom Inventory; CAMS = Collaborative Assessment and Management of Suicidality; CBS = Cyberbullying Scale; CBSS = Cyberbullying Sensibility Scale; CDI = Children Depression Inventory; CVS = Cybervictimisation Scale; DERS = Difficulties in Emotion Regulation Scale; DGAS = Digital Game Addiction Scale; DM = Direct Messaging; DSHI = Deliberate Self-Harm Inventory; EMA = Ecological Momentary Assessment; FOMO = Fear of missing out; h/day = Hours per day; HAM-A = Hamilton Anxiety Rating Scale; HAM-D = Hamilton Rating Scale for Depression; IAD = Internet Addiction Disorder; IAS = Internet Addiction Scale; IAT = Young’s Internet Addiction Test; ID = Internalising Disorder; IGD = Internet Gaming Disorder; K-SADS-PL = Kiddie Schedule for Affective Disorders and Schizophrenia for School-Age Children-Present and Lifetime version; M = Mean; MDD = Major Depressive Disorder; MEBMI = Mean Estimated Body Mass Index; MIDI = Minnesota Impulsive Disorder Inventory; PHQ = Patient Health Questionnaire; PIU = Problematic Internet Use; PIU-SF-6 = Problematic Internet Use Short Form 6; PMPUS = Problematic Mobile Phone Usage Scale; PUI-SQ = Screening Questionnaire for Problematic Use of the Internet; R = Age Range; RCADS = Children's Anxiety and Depression Scale; SA = Suicide Attempt; SAS-SV = Smartphone Addiction Scale-Short Version; SD = Standard Deviation; SDQ = Strengths and Difficulties Questionnaire; SEG = Socio-economic Group; SHBQ = Self-Harm Behavior Questionnaire; SI = Suicidal Ideation; SIQ-Jr = Suicide Ideation Questionnaire – Junior; SMDS = Social Media Use Disorder Scale; SMS = Social Media Sites; SNS = Social Network Sites; YDQ = Young Diagnostic Questionnaire for Internet Addiction; YIAT = Young Internet Addiction Test; Y-PSC = Youth Pediatric Symptom Checklist.
