# Supplementary material for: Review: Digital experiences and their impact on the lives of adolescents with pre‐existing anxiety, depression, eating and nonsuicidal self‐injury conditions – a systematic review
Source: Child Adolesc Ment Health. 2022 Dec 7;28(1):22–32. doi: 10.1111/camh.12619 (PMC10108198; doi:10.1111/camh.12619)
Supplement: Supplementary file 2 — Appendix S1. Search Terms. [file CAMH-28-22-s002.docx]

**Appendix S1.** Search terms

*Mental health terms*:

- Eating disorders: eating disorder, appetite disorder, binge eating disorder, anorex*, bulim*, hyperphagia, pica, rumination regurgitation disorder, orthorex*, avoidant restrictive food intake disorder, overeating, compulsive eating, compulsive vomiting, food addiction, pro-Ana, pro-eating, body image,
- Self-harm: non-suicidal, self?injur*, self?injurious behavio*, self-mutilat*, automutilat*, self-destructive behavio*, self-damag*, self-harm*, self-destruct*, self-hurt, self-violen*, self-wound*, self-inflicted injur*, selfinflicted wounds,
- Depression or anxiety: internal* disorder, depress*, worry, anxiety, mental health, emotion* disorder.

*Digital engagement terms*:

- digital*, mobile*, internet, online, social media, cyber*, app, technolog* comput*, gaming, social networking sites, browsing history, hashtag, Instagram, Facebook, Reddit, Twitter, TikTok, YouTube, WhatsApp, Tumblr, instant messag*, Snapchat, smartphone, web forum, web chat, internet forum

*Population terms*:

- child*, youth, teen*, “adolescen*, pediatric*.
